# Simulations of NPC1(NTD):NPC2 Protein Complex Reveal Cholesterol Transfer Pathways

**DOI:** 10.3390/ijms19092623

**Published:** 2018-09-04

**Authors:** Milan Hodošček, Nadia Elghobashi-Meinhardt

**Affiliations:** 1 National Institute of Chemistry, Hajdrihova 19, 1001 Ljubljana, Slovenia; milan@cmm.ki.si; 2 Department of Chemistry and Biochemistry, Freie Universität Berlin, 14195 Berlin, Germany

**Keywords:** Niemann-Pick type C disease, NPC1, NPC2, computations, simulations, cholesterol ligand, ligand transfer, binding free energy, interface stability, molecular dynamics, molecular modeling

## Abstract

The Niemann Pick type C (NPC) proteins, NPC1 and NPC2, are involved in the lysosomal storage disease, NPC disease. The formation of a NPC1–NPC2 protein–protein complex is believed to be necessary for the transfer of cholesterol and lipids out of the late endosomal (LE)/lysosomal (Lys) compartments. Mutations in either NPC1 or NPC2 can lead to an accumulation of cholesterol and lipids in the LE/Lys, the primary phenotype of the NPC disease. We investigated the NPC1(NTD)–NPC2 protein–protein complex computationally using two putative binding interfaces. A combination of molecular modeling and molecular dynamics simulations reveals atomic details that are responsible for interface stability. Cholesterol binding energies associated with each of the binding pockets for the two models are calculated. Analyses of the cholesterol binding in the two models support bidirectional ligand transfer when a particular interface is established. Based on the results, we propose that, depending on the location of the cholesterol ligand, a dynamical interface between the NPC2 and NPC1(NTD) proteins exists. Structural features of a particular interface can lower the energy barrier and stabilize the passage of the cholesterol substrate from NPC2 to NPC1(NTD).

## 1. Introduction

The Niemann Pick type C (NPC) proteins, NPC1 and NPC2, have received a great deal of attention in recent years, not only as they are involved in the lethal hereditary NPC disease, but also as the NPC1 protein has been identified as being necessary for Ebola and Marburg virus infection [1,2]. Specifically, viral infection occurs when the virus glycoprotein (GP) binds to the NPC1 protein. Cells deficient in the NPC1 protein are protected from infection. On the other hand, mutations in either NPC1 or NPC2 can lead to an accumulation of cholesterol and lipids in the late endosomal (LE)/lysosomal (Lys) compartments, the primary phenotype of the Niemann-Pick type C (NPC) disease. Thus, understanding structural features of the NPC1 and NPC2 binding domains is the first step in developing therapeutic treatments for the NPC disease as well as designing inhibitors against Ebola and Marburg viruses.

The exact role of NPC2 is not known, but a model has been proposed in which NPC2 binds cholesterol after receptor-mediated endocytosis of low-density lipoprotein (LDL) [3]. After binding, NPC2 may transport cholesterol to the membrane-bound NPC1 protein (1278 amino acids), where cholesterol can be transferred to the N-terminal domain (NTD) of NPC1. From there, cholesterol is likely transferred to NPC1’s membrane domain for subsequent biochemical processing [3]. This mechanism of cholesterol transfer is supported by X-ray crystallography that shows the cholesterol’s isooctyl side chain buried deep inside the hydrophobic pocket of NPC2 with its 3β-hydroxyl group exposed at the protein’s surface [4]. On the other hand, NPC1(NTD) binds cholesterol in the opposite orientation: the 3β-hydroxyl group is buried in the binding pocket while the cholesterol’s isooctyl side chain is surface exposed [5]. These findings led to the proposal of a “hydrophobic hand-off” or sliding model, in which cholesterol is transferred between NPC2 and NPC1(NTD) without exposure to water [6].

Experimental evidence supports the sliding model of cholesterol transfer between NPC2 and NPC1(NTD). Surface plasmon resonance experiments, together with affinity chromatography, have revealed that the second luminal loop-domain of NPC1 maintains the position of NPC2 at the binding interface to facilitate cholesterol transfer [7]. Biochemical assays have shown that deletion of the NTD (residues 25–257) of NPC1 abolish more than 90% of cholesterol transfer from NPC2 to NPC1(NTD) [8]. A patch of primarily three residues (M79, V81, and V83) on the surface of NPC2 have been found to be essential for transfer of cholesterol from NPC2 to NPC1(NTD) [6]. Similarly, critical mutations (L175Q and L176Q) at the surface of NPC1(NTD) abolish cholesterol transfer activity [6].

Nonetheless, a direct interaction between NPC1(NTD) and NPC2 has thus far not been demonstrated. The geometry of a putative NPC1(NTD)–NPC2 protein complex was proposed by the group of Brown and Goldstein at UT Southwest Medical Center in Texas [3,6]. Their structure was assembled from rigid body alignment using the information from point mutation experiments identifying amino acids involved at the potential interface between the two individual proteins [3,6]. Using this structure, which we refer to as the “Texas model”, Estiu et al. performed MD simulations and *in silico* mutational analyses [9]. Using the Texas model, we also examined cholesterol isomerization inside the NPC1 and NPC2 binding pockets, proposing a model for cholesterol transfer between the binding pockets of the NPC1(NTD)–NPC2 protein complex [10].

**Figure 1:**
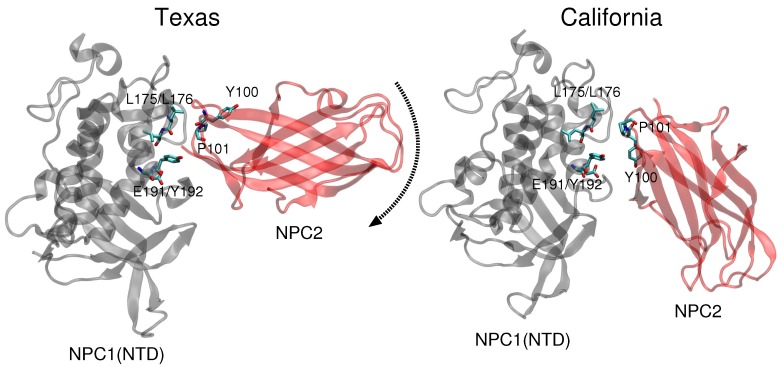
The NPC1(NTD) (gray)–NPC2 (red) complex is shown for the Texas model based on alignment proposed in Ref. [3,6] and for the California model, based on the structure proposed in Ref. [11]. The black dotted arrow indicates the rotation direction that transforms the position of the NPC2 protein in the Texas model to its position in the California model. The California interface results in Y100 of NPC2 being in closer proximity to E191/Y192 of NPC1.

More recently, Li et al. at Stanford University presented a crystal structure (2.4 Å resolution) of the soluble NPC2 protein bound to the NPC1 middle lumenal domain (MLD) (residues 374–620) [11]. Docking of this complex on a low resolution (4.43 Å) cryo-EM structure of full-length NPC1 protein, Li et al. proposed a new putative NPC1(NTD)–NPC2 interface, which we refer to as the “California model”, that reveals a somewhat different interface but a similar cholesterol transfer tunnel as proposed in the Texas model. In the California model [11], the angle between NPC2 and NPC1(NTD) is more acute than in the Texas model [9] (see Figure 1). In the California model, NPC2’s P120 (corresponding to P101 in PDB 2HKA from Ref. [4]) is close to NPC1’s NTD K179 (C${}_{\alpha }$-C${}_{\alpha }$ separation 4.3 Å) and D180 (C${}_{\alpha }$-C${}_{\alpha }$ separation 5.2 Å) [11]. In comparison, in the Texas model, P120 is farther away from NPC1’s NTD K179 (C${}_{\alpha }$-C${}_{\alpha }$ separation 8.1 Å) and D180 (C${}_{\alpha }$-C${}_{\alpha }$ separation 10.0 Å) [9].

We revisited the problem of the NPC1(NTD)–NPC2 interface and analyzed the Texas and California models of the protein–protein complex with respect to cholesterol transfer pathways. We compared the binding energies associated with each of the binding pockets for the two models, and we computed energy barriers corresponding to cholesterol transfer between the NPC binding pockets for each of the two proposed geometries. Based on our analyses, we propose that, depending on the location of the cholesterol ligand, a dynamical interface between the NPC2 and NPC1(NTD) proteins presents structural advantages that can lower the energy barrier and stabilize the passage of the cholesterol substrate. Understanding the atomic details of the interface between the water-soluble NPC2 protein and the membrane-bound NPC1 protein may thus serve as a foundation for therapeutic protein engineering.

## 2. Results and Discussion

Comparison of the two constructed models (Figure 1) highlights the difference in relative angle between the NPC1 and NPC2 protein subunits and the alignment of potentially functional amino acids.

**Figure 2:**
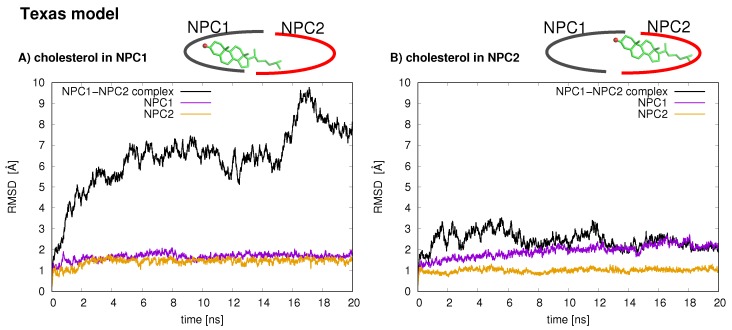
Protein backbone atom RMSD values for the NPC1(NTD)–NPC2 complex of the Texas model based on the structure proposed in Ref. [3,6] is shown: (**A**) with cholesterol in the NPC1 binding pocket; and (**B**) with cholesterol in the NPC2 binding pocket. Black line corresponds to the RMSD for the total protein–protein complex, purple line corresponds to RMSD of NPC1 protein, and orange line corresponds to RMSD of NPC2 protein.

**Figure 3:**
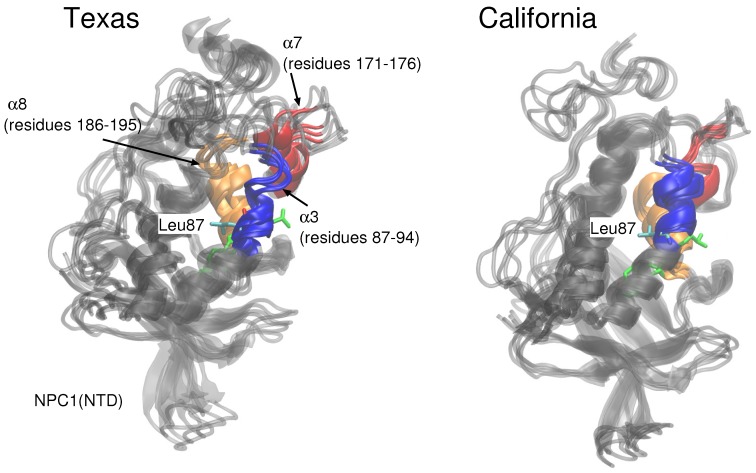
Conformational changes in α3 (blue), α7 (red), and α8 (orange) helices are shown for snapshots throughout the 20 ns MD simulation for the Texas model and for the California model. Leu87, labeled in the figures, interacts with the cholesterol isooctyl tail in the Texas model.

The stabilities of the two protein–protein complexes were checked first by examining the RMSD of protein backbone atoms of the complexes and the individual protein subunits over the 20 ns simulation times. For each model, the cholesterol was simulated in each binding pocket, resulting in four MD trajectories, each 20 ns in length. The RMSD of the Texas model (black line is Figure 2) when cholesterol is located in the NPC1 binding pocket rapidly increases and peaks around 18 ns at close to 10 Å. Estiu et al. also report large RMSD values for their complex, both in an apo state and when cholesterol is located in the NPC1 binding pocket [9]. In particular, for the case in which cholesterol is located in the NPC1 binding pocket, they observed significant shifts in the positions of α-helices 3 (residues 87–94), 7 (residues 171–176), and 8 (residues 186–195) [9] (loop designation according to Ref. [3]). Estiu et al. attributed the conformational changes to the interaction of Leu87 with the isooctyl chain of the cholesterol. Similar shifts were observed in our simulations with cholesterol in the NPC1 binding pocket, particularly α7 (Figure 3 Texas model, red helix) demonstrates a large conformational change; Leu87 interacts with the cholesterol ligand. When cholesterol is located in the NPC2 binding pocket, the same model exhibits RMS differences that fluctuate between 2 and 3 Å, also in agreement with the behavior observed by Estiu et al. [9]. For each binding scenario, the RMSD values of the individual protein subunits were also checked; both NPC1 (purple line) and NPC2 (orange line) demonstrate reasonable structural integrity with RMSD values between 1 and 2 Å.

**Figure 4:**
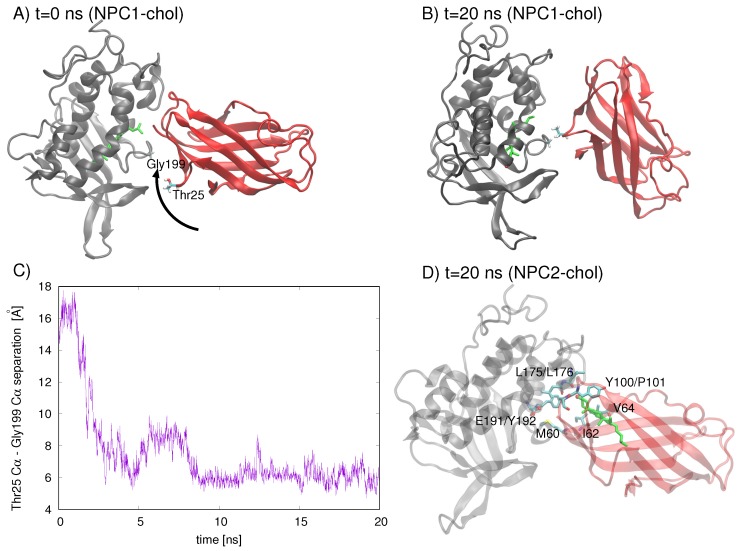
The relative orientation of the Texas model NPC1(NTD)-NP2 subunits with cholesterol in the NPC1 binding pocket (gray cartoon representation) at (**A**) *t* = 0 ns and (**B**) *t* = 20 ns highlights the rotation of the subunits with respect to each other. (**C**) The rotation brings two protruding loops, with Thr25 of NPC2 (red cartoon representation) and Gly199 of NPC1, into close proximity (Thr25 C${}_{\alpha }$–Gly199C${}_{\alpha }$ decreases from ∼16 Å to ∼6 Å). (**D**) The relative orientation of the Texas model NPC1(NTD)-NP2 subunits (shown here in transparent cartoon representation for clarity) with cholesterol (green stick) in the NPC2 binding pocket at *t* = 20 ns preserves the interface interactions between NPC1 (L175, L176, E191, Y192) and NPC2 (M60, I62, V64, Y100, P101).

An analysis of residues involved in the Texas model’s interface at *t* = 0 ns and *t* = 20 ns indicates that the NPC2 protein rotates with respect to NPC1, increasing the separation between the initial interface separation. The rotation brings Gyl199 of NPC1 into close contact (∼5 Å) with Thr25 of NPC2 (Figure 4C). On the other hand, when cholesterol is in the NPC2 binding pocket of the Texas model, the interface is still intact at *t*= 20 ns, with the cholesterol hydroxy group protruding into the interface space (Figure 4D). Particularly M60, I62, and V64 of NPC2 (corresponding to M79, V81, and V83, respectively, in Ref. [6]), which have been identified in alanine mutagenesis studies as being critical for preserving transfer function [6], maintain their position in the interface.

**Figure 5:**
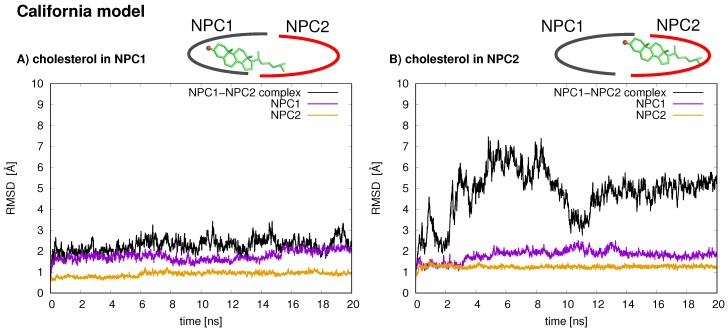
Protein backbone atom RMSD values for the NPC1(NTD)–NP2 complex of the California model. Ref. [11] is shown for: (**A**) with cholesterol in the NPC1 binding pocket; and (**B**) with cholesterol in the NPC2 binding pocket. Black line corresponds to the RMSD for the total protein–protein complex, purple line corresponds to RMSD of NPC1 protein, and orange line corresponds to RMSD of NPC2 protein.

In the California model, the protein–protein complex exhibits relatively small (2–3 Å) RMSD values when the cholesterol ligand is in the NPC1 binding pocket (black line Figure 5A), as compared to the situation with cholesterol in the NPC2 binding pocket (black line Figure 5B), which shows fluctuations around 6–7 Å. In the California model, the relative structural integrity of the NPC1 protein with cholesterol in its binding pocket, compared to the Texas model, can be observed in Figure 3. All three helices, α3 (blue), α7 (red), and α8 (orange), show minimal changes throughout the 20 ns MD simulation. These differences suggest that the relative position of and the angle between the two protein subunits may influence the interface stability, depending on the location of cholesterol. In the Texas model, the complex is more stable with cholesterol located in NPC2 while, in the California model, the reverse is true. Based on these results, one could speculate that the two proteins undergo dynamical rearrangement depending on the location of the cholesterol. One possible interpretation is that the Texas model represents better a stable binding interface before the transfer of cholesterol when it is still in the binding pocket of NPC2. The California model represents a more stable conformation after ligand transfer when cholesterol is located in NPC1(NTD).

To check whether the geometry of the protein–protein complex affects binding energy, we performed solvation energy analyses of the ligand binding strengths using the GBSW approach. Computations indicate that in the Texas model, the NPC2/chol complex is energetically stabilized relative to the complex with cholesterol in the NPC1 binding pocket by approximately −1.48 kcal/mol. In the California model, the corresponding binding energy stabilization is −2.91 kcal/mol. The cholesterol binding behavior of NPC2 differs from that of NPC1(NTD) in in vitro studies as well [12]. NPC2 binds cholesterol rapidly and reversibly at 4 ${}^{{}^{\circ}}$C as well as at 37 ${}^{{}^{\circ}}$C, behaving—according to the authors—as a “typical” receptor [12]. NPC1(NTD), on the other hand, binds differently, slowly binding cholesterol at 4 ${}^{{}^{\circ}}$C and not releasing the lipid at this temperature; these on-off rates are accelerated at 37 ${}^{{}^{\circ}}$C [12]. The authors state that this binding behavior is indicative of a closed conformation of NPC1(NTD) that is opened in the presence of NPC2. It should be noted that experimental binding and transfer rates are obtained via assays that are conducted over a period of several minutes (including incubation and elution); the experimentally measured values are likely an underestimate of the true transfer rates [12]. In the context of this computational study, the experimentally measured values serve only as a qualitative comparison for the behavior of the two proteins that have been simulated here for hundreds of nanoseconds.

**Figure 6:**
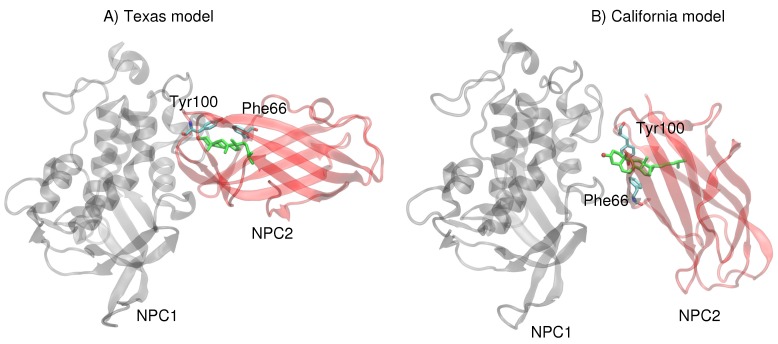
(**A**) In the Texas model, the cholesterol ring system is engaged in π–π interactions with Phe66 and Tyr100 of NPC2. (**B**) In the California model, Phe66 and Tyr100 of NPC2 point downward and away from the cholesterol ligand.

Next, more rigorous free energy perturbation calculations were carried out, using MD simulations with explicit water, to compare the relative free energies of binding of cholesterol in each of the binding pockets for both models. In the Texas model, the free energy of binding in NPC1 was calculated to be −60.0 kcal/mol (±3.1 kcal/mol), and in NPC2 −60.9 kcal/mol (±3.1 kcal/mol). The results are qualitatively similar to the GBSW results, suggesting that the binding pockets in the Texas model indicate no significant free energy gain for cholesterol binding in the NPC1 binding pocket. In the California model, the free energy of binding in NPC1 is somewhat less favorable (−52.1 ± 2.9 kcal/mol) compared to the binding in the NPC2 pocket (−54.4 ± 2.9 kcal/mol). Overall, the California model appears to give weaker cholesterol binding interactions than does the Texas model. Reasons for this tighter binding in the Texas model may be found in the interactions particularly of the 3β-hydroxy end that is involved in π–π interactions with Phe66 and Tyr100 of NPC2 [13]. In the Texas model, the cholesterol is engaged with Tyr100 and Phe66 which are positioned above the ligand (Figure 6A). In the California model, on the other hand, both Tyr100 and Phe66 are pointing downward and away from the cholesterol ligand (Figure 6B). Therefore, the interface of the Texas model may result in more favorable hydrophobic environment, stabilizing the ligand position in the NPC2, as we observed in the RMSD behavior discussed earlier (see Figure 2).

In vitro data indicate that cholesterol transfer between NPC1 and lipid bilayers facilitated by NPC2 is bidirectional [12]. Our computations show similar ligand binding strengths for NPC1 and NPC2, which is particularly true for the Texas model. Hence, a bidirectional transfer of cholesterol would be feasible. On the other hand, the orientation of the California model, in which the long axis of the binding pockets of the two protein subunits are more closely aligned, may yield a lower barrier for ligand transfer. Again, our results support the notion of a dynamical interface in which the alignment of the NPC1(NTD) and NPC2 proteins changes depending on the location of the ligand. The pocket alignment of the Texas model may reflect a recognition pose of protein–protein docking, during which the interface is being established. Bidirectional ligand transfer may be possible in the Texas alignment, whereas the California alignment may favor the unidirectional transfer of cholesterol from NPC2 to NPC1.

**Figure 7:**
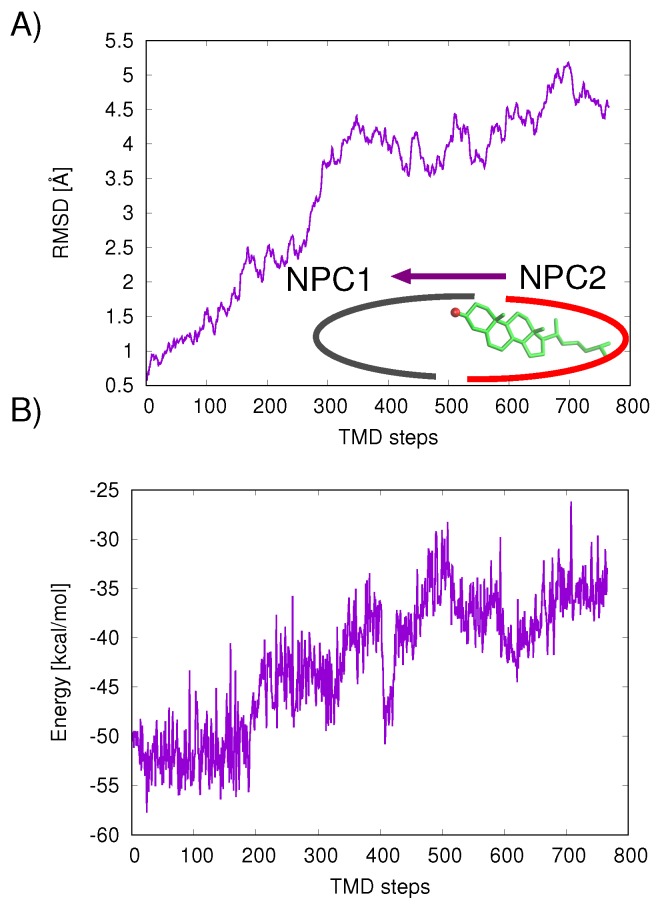
For the Texas model, TMD results of cholesterol transfer in the direction NPC2→NPC1 (purple line) are analyzed: (**A**) protein backbone RMS differences (Å); and (**B**) cholesterol–protein potential energies associated with each TMD path are plotted for the TMD path.

**Figure 8:**
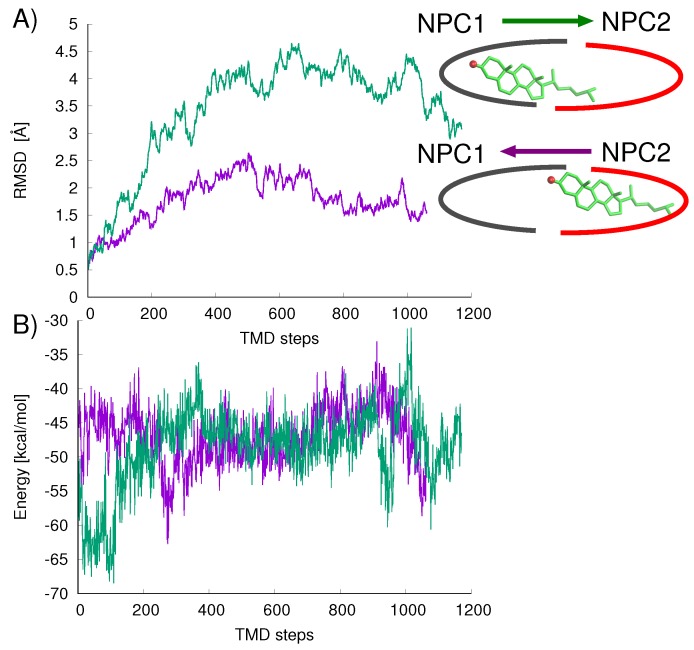
TMD results of cholesterol transfer based on the California model in the direction NPC2→NPC1 (purple line) shows: (**A**) overall lower protein backbone RMS differences (Å) compared to the opposite direction, NPC1→NPC2 (green line); (**B**) cholesterol–protein potential energies associated with each TMD path are plotted for both transfer directions.

The mechanism of ligand transfer was studied next for both the Texas and California models using TMD simulations. For the Texas model, simulations of cholesterol transfer in the direction NPC2→NPC1 indicated instabilities at the protein–protein interface, evidenced by the increase in protein backbone RMSD values (Å) (Figure 7A) and increase in cholesterol–protein potential energies (Δ*E* = +16.9 kcal/mol) (Figure 7B). For the California model, relatively lower RMS differences were measured for the cholesterol transfer path NPC2→NPC1 (purple line in Figure 8A). For the opposition direction, NPC1→NPC2, the California model shows higher protein backbone RMS differences along the path (green line in Figure 8A). The cholesterol–protein potential energy changes associated with the ligand transfer (Figure 8B) are overall more favorable for the transfer of cholesterol in the NPC2→NPC1 direction (Δ*E* = −12.6 kcal/mol) compared to the transfer in the opposite direction NPC1→NPC2 (Δ*E* = +8.3 kcal/mol).

**Figure 9:**
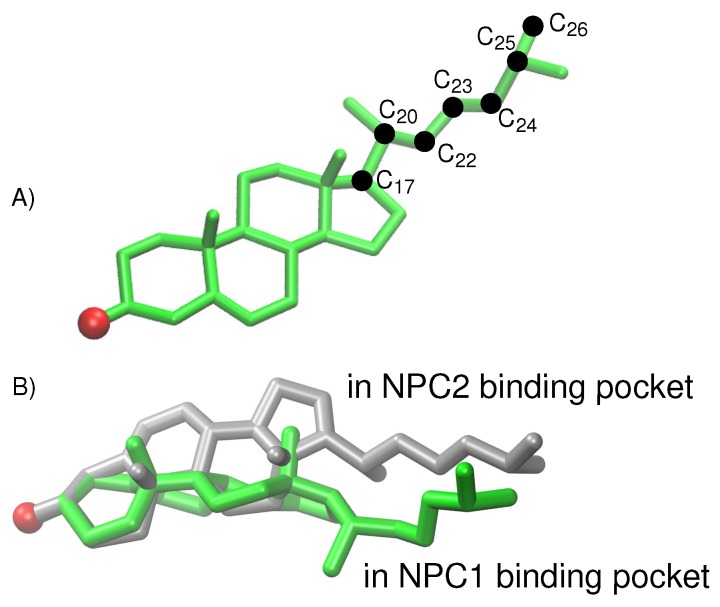
(**A**) Model of cholesterol with carbon atoms (green) involved in tail isomerization indicated with black dots (oxygen atom shown in red). (**B**) Comparison of the cholesterol conformations in the binding pockets of the California model, NPC1(NTD) (green) and the NPC2 (gray).

**Figure 10:**
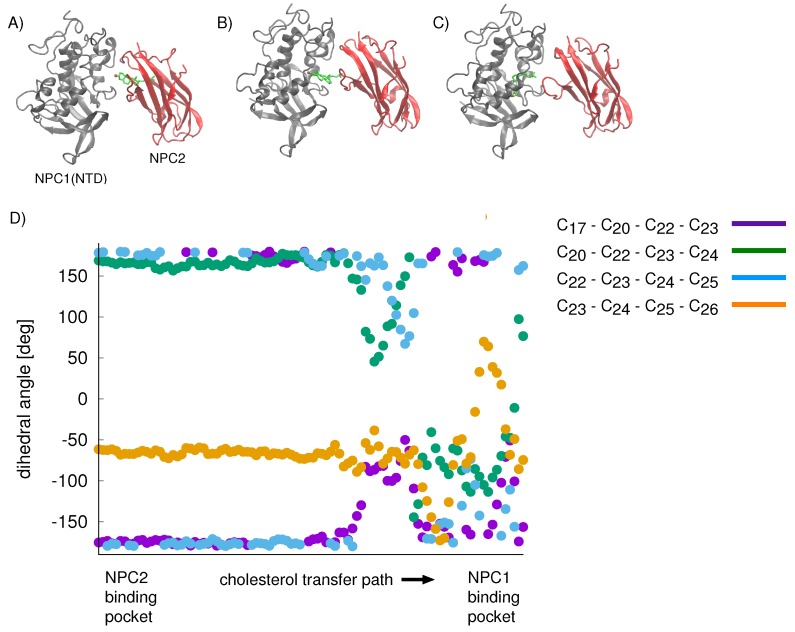
The cholesterol transfer path involves: (**A**) cholesterol leaving the binding pocket of the NPC2 protein; (**B**) crossing a transition state; and (**C**) entering the binding pocket of the NPC1(NTD). (**D**) For cholesterol transfer based on the California model, the conformation of the cholesterol tail was analyzed during the transfer from the NPC2 binding pocket to the NPC1(NTD) binding pocket. Four dihedral angles were calculated: C${}_{17}$-C${}_{20}$-C${}_{22}$-C${}_{23}$ (purple), C${}_{20}$-C${}_{22}$-C${}_{23}$-C${}_{24}$ (green), C${}_{22}$-C${}_{23}$-C${}_{24}$-C${}_{25}$ (blue), and C${}_{23}$-C${}_{24}$-C${}_{25}$-C${}_{26}$ (orange).

For the cholesterol transfer modeled using the California model, we computed the dihedral angles of the cholesterol tail (Figure 9A) over the course of the TMD path (Figure 10D). The coupled twisting of C${}_{17}$-C${}_{20}$-C${}_{22}$-C${}_{23}$ and C${}_{20}$-C${}_{22}$-C${}_{23}$-C${}_{24}$ dihedral angles is similar to the behavior that was observed previously in our study of cholesterol isomerization inside the binding pockets of NPC1 and NPC2 [10]. Inside the NPC2 binding pocket (gray model in Figure 9B), the dihedral angle of cholesterol’s C${}_{17}$-C${}_{20}$-C${}_{22}$-C${}_{23}$ atoms is <$-150^{{}^{\circ}}$ (purple dots in Figure 10D). The C${}_{22}$-C${}_{23}$-C${}_{24}$-C${}_{25}$ dihedral angle (blue dots in Figure 10D) takes on similar values, as observed previously [10]. During the handover of the ligand at the protein–protein interface (Figure 10B), the C${}_{17}$-C${}_{20}$-C${}_{22}$-C${}_{23}$ dihedral angle approaches −50${}^{{}^{\circ}}$, before relaxing to <$-150^{{}^{\circ}}$ inside the NPC1(NTD) binding pocket (green model in Figure 9B).

## 3. Methods

### 3.1. Construction of Model

As in our previous work [10], the NPC1(NTD) and NPC2 protein components were modeled individually based on the higher-resolutions structures of Kwon (Ref. [3]) and Xu (Ref. [4]), respectively. For this purpose, the wild-type NPC1(NTD) protein bound to cholesterol was constructed based on the 3GKI (1.80 Å) crystal structure [3]. The NPC1(NTD) contains amino acid residues 23–247 and 100 water molecules [3]. For NPC2, the structure was based on the PDB structure 2HKA (1.81 Å, bovine) containing the amino acid residues 1–130 and 110 water molecules; the cholesterol sulfate in the crystal structure was converted to cholesterol [4]. We would like to point out that our numbering of NPC2 residues begins after the 19-amino acid signal sequence, according to Ref. [4,14], i.e., residues M79, V81, and V83 cited in Ref. [6] for human NPC2 sequence correspond to M60, I62, and V64, respectively, in our model. Hydrogen atoms were added using H-build from CHARMM [15]. The N- and C-termini were capped with neutral groups CH${}_{3}$–CO– and methyl acetate –NH–CO–OCH${}_{3}$, respectively. Based on the crystal structures, we constructed twelve disulfide bonds using CHARMM, nine in NPC1(NTD) (Cys25–Cys74, Cys31–Cys42, Cys63–Cys109, Cys75–Cys113, Cys97–Cys238, Cys100–Cys160, Cys177–Cys184, Cys227–Cys243, and Cys240–Cys247) and and three in NPC2 (Cys8–Cys121,Cys23–Cys28,and Cys74–Cys80).

Next, we modeled two NPC1–NPC2 protein–protein complexes, one with cholesterol in the NPC1 binding pocket and the other with cholesterol in the NPC2 binding pockets, by positioning the two individual protein crystal structures with their respective water molecules using the interfaces of the Texas [9] and California [11] models, respectively, as templates. In the California model, to align the NPC1(NTD) and NPC2 interfaces, we followed the protocol described in Ref. [11]. Namely, we first positioned the NPC1-MLD structure (PDB 5KWY, 2.41 Å resolution [11]) onto the lower resolution (4.43 Å) cryo-EM structure of the MLD of NPC1 (PDB 3JD8) [8]. Next, we overlapped the two protruding loops (residues 422–425 on loop1 and residues 501–503 on loop2) of the MLDs of NPC1 to align the cholesterol binding pockets. With the interface thus established, we superimposed the higher resolution NPC1 (PDB 3GKI) and NPC2 (PDB 2HKA) protein structures, with their corresponding cholesterol ligands and water molecules, onto the complex and saved the coordinates.

Water molecules from one PDB structure overlapping with water molecules from the other PDB structure were deleted (13 molecules occurring near the interface of the two crystal structures), resulting in a total of 197 water molecules from the crystal structures. Water molecules in the protein binding pockets were not affected by this protein–protein modeling and remained unchanged. The total number of atoms present in the final California and Texas models was 6139. For both structures, the cholesterol was modeled either in the NPC1 binding pocket, NPC1(NTD)[chol]–NPC2, or in the NPC2 binding pocket, NPC1(NTD)–NPC2[chol].

### 3.2. Geometry Optimization

The initial geometries of the NPC1(NTD)–NPC2 complexes with cholesterol were optimized first with 50 steps of steepest descent (SD) energy minimization, followed by 50,000 adopted basis Newton–Raphson (ABNR) [15] steps to remove any close contacts. Next, the protein–protein complex was solvated in a rectangular water box. For the Texas model, the box has dimension 100 Å × 75 Å × 55 Å, containing a total of 43,891 atoms (12,794 explicit TIP3 [16] water molecules); for the California model, the box has dimension 105 Å × 95 Å × 65 Å containing 68,741 atoms (21,076 explicit TIP3 water molecules [16]). The solvated proteins were relaxed with an additional 50 SD steps, followed by 100 ABNR steps, before 100 ns equilibration was performed at 310 K. The simulation system was treated using the all-atom CHARMM36 parameter set for the protein [17] and the TIP3P model for water molecules [16]. The force field parameters for cholesterol were developed and optimized at the CCSD(T)/cc-pVQZ level and included in the current CHARMM parameter set [18,19].

### 3.3. Energy Path Calculations

To compute an initial pathway for the cholesterol transfer between the binding pockets of the two modeled NPC protein complexes, targeted molecular dynamics (TMD) [20], as implemented in CHARMM [21], was used. In TMD, a transition pathway is calculated from a starting conformation to the target state by applying a single time-dependent holonomic constraint based on the (mass-weighted) RMSD between the two conformations [20]:

(1) $$\phi \left(\vec{X},t\right)=\left(\sum_{\left(i=1\right)}^{N}m_{i}{\left|{\vec{x}}_{i}\left(t\right)-{\vec{x}}_{i,F}\right|}^{2}/\sum_{i=1}^{N}m_{i}\right)-\zeta^{2}\left(t\right)=0$$

where *N* is the number of atoms subjected to the constraining condition, Equation (1), ${\vec{x}}_{i,F}$ is the position of atom *i* in the target conformation, ${\vec{x}}_{i}\left(t\right)$ is the position of the atom *i* at time *t*, ζ(t) is the selected mass-weighted RMSD between the system and the target structure at time *t*, $m_{i}$ is the mass of atom *i*, and $\vec{X}=\left\{{\vec{x}}_{1},{\vec{x}}_{2},\ldots ,{\vec{x}}_{N}\right\}$. At each step of the MD simulation, the system evolves according to the potential energy function. The constraint forces, ${\vec{F}}_{i}^{c}=\delta \phi /\delta {\vec{x}}_{i}$, then perturb the system to satisfy Equation (1).

For our TMD path calculations, a selection of protein and cholesterol atomic positions were considered in initial and target structures. In addition to the positions of cholesterol atoms, for the path from NPC2 to NPC1, the atomic positions of three NPC1 residues (W27, Q79, and N86) were selected for targeting; for the path NPC1 to NPC2, the atomic positions of three NPC2 residues (V20, F66, and V96) were chosen for targeting. An initial RMSD distance of 11.7 Å was incrementally (0.00001 Å) reduced until a target RMSD distance of 0.000001 Å was reached. The trajectory was sampled at 310 K with 1 fs time steps for a total of 1.5 ns.

### 3.4. Molecular Dynamics

The solvated protein–protein complexes of the Texas and California models were simulated with molecular dynamics (MD) for 20 ns at 310 K. The MD simulations used an integration time step of 2 fs and SHAKE algorithm was used to constrain all bonds involving hydrogen atoms [22]. To simulate a continuous system, periodic boundary conditions were applied. Electrostatic interactions were summed with the Particle Mesh Ewald method [23] (grid spacing ∼1.3 Å; fftx 80, ffty 72, fftz 48). A nonbonded cutoff of 16.0 Å was used, and Heuristic testing was performed at each energy call to evaluate whether the non-bonded pair list should be updated.

### 3.5. Solvation Energies

To calculate the relative stabilities of the substrate-bound NPC1 and NPC2 proteins, the Generalized Born Simple Switching (GBSW) module in CHARMM was used. For the simulation of both binding scenarios (NPC1[chol]–NPC2 and NPC1–NPC2[chol]) for both Texas and California models, 5000 coordinate frames were extracted from each MD over 20 ns. Three sets of coordinates (substrate-protein, apo protein, and substrate) were extracted then from a single MD run, and the GBSW solvation free energy was calculated for each extracted frame and then averaged. The dielectric constant of the solvent set to 80 and inside the protein volume to 4; the nonpolar surface tension coefficient was set to 0.0072 kcal/(mol × Å${}^{2}$).

### 3.6. Perturbation Free Energy Calculations

The cholesterol binding free energy was calculated for the binding pockets of Texas and California models. For these slow growth free energy computations, the PERT module of CHARMM was employed [15]. The electrostatic and van der Waals forces of the cholesterol substrate were slowly turned off using a switching parameter λ over a series of twenty steps, and the free energy was calculated as a function of the switching parameter. For each cholesterol in a binding pocket, a total of 150 ps was simulated five times.

## 4. Conclusions

Here, we have compared two similar binding interfaces of the NPC1(NTD) and NPC2 proteins. MD simulations indicate that the position of the cholesterol in the protein–protein complex affects the stability of the interface, as evidenced by the RMSD values of the models over 20 ns simulation time. In the Texas model, the NPC1(NTD)–NPC2 complex is unstable with cholesterol in the NPC2 binding pocket, whereas, in the California model, the complex is unstable when cholesterol is in the NPC1 binding pocket. As a bidirectional cholesterol transfer has been observed in cholesterol transfer assays, our findings may support the existence of two feasible docking interfaces. According to this hypothesis, the Texas interface may correspond to the situation after cholesterol has been transferred, whereas the California interface is more stable with cholesterol in the NPC2 binding pocket. Free energy analyses of the cholesterol binding strengths in the binding pockets of the two models present arguments that support bidirectional ligand transfer particularly in the Texas model. TMD simulations with the California model have provided us with plausible minimum energy paths connecting the binding pockets and indicate that cholesterol transfer from NPC2 to NPC1 is energetically favorable.

One mechanistic explanation for the difference in proposed protein–protein conformations may be that, in the Texas model, the residues involved in recognition are used to establish the interface. In this conformation, cholesterol transfer may take place. As the protein domains realign according to the California model, forward transfer of cholesterol, e.g., in the direction NPC2→NPC1(NTD), is secured. In this pose, the free energy gain is realized and cholesterol is positioned for subsequent insertion into the lysosomal membrane.

As a direct interaction between NPC2 and NPC1(NTD) has thus far not been detected, one may speculate that the isolated NTD may be insufficient to establish a stable interaction. In fact, domain C of NPC1’s MLD which links the transmembrane domain to the NTD, may be needed to establish the correct binding pose for NPC2 [8]. Indeed, 28 NPC disease mutations map to NPC1’s domain C [24]. Furthermore, the NTD also may require specific interactions with the C-terminal domain (CTD) (residues 861-1083), as disruption of this interface has been shown to block cholesterol transfer from the late endosome to ER [25]. Our ongoing investigations are therefore directed at constructing a more complete NPC1 model that includes several domains (NTD, MLD, and CTD) and simulating this with the NPC2 to analyze additional atomic interactions required for stabilizing the protein–protein interface. These efforts are greatly supported by the emergence of higher resolution X-ray crystallography data as well as cryo-EM data elucidating atomic detail of the NPC1 domains. Our simulations have been carried out with bovine NPC2 which has a highly conserved sequence homology to human NPC2. Nonetheless, repeating our simulations with human NPC2 would be an interesting and worthwhile comparison of binding interactions.

Ultimately, our mechanistic understandings of the interactions between NPC1 and NPC2 may help rationalize the severity of different NPC disease genotypes. This knowledge is also useful for understanding NPC1’s interactions with other proteins found in the lysosomal, including the Ebola GP. Interestingly, at pH = 6, the Ebola GP binds 80× tighter to the NPC1 protein than to NPC2 in vitro [8]. As our understanding of the molecular interactions involved in protein–protein binding increases, the reasons for this competitive binding may become clearer.

## Abbreviations

The following abbreviations are used in this manuscript:

NPC	Niemann-Pick type C
NPC1	NPC protein 1
NPC2	NPC protein 2
MD	Molecular dynamics
TMD	Targeted molecular dynamics
NTD	N-terminal domain
MLD	Middle luminal domain
CTD	C-terminal domain
